# Location and Identification on Chromosome 3B of Bread Wheat of Genes Affecting Chiasma Number

**DOI:** 10.3390/plants11172281

**Published:** 2022-08-31

**Authors:** Benoit Darrier, Isabelle Colas, Hélène Rimbert, Frédéric Choulet, Jeanne Bazile, Aurélien Sortais, Eric Jenczewski, Pierre Sourdille

**Affiliations:** 1 UMR 1095 Genetics, Diversity and Ecophysiology of Cereals, 5, INRAE–Université Clermont-Auvergne, Chemin de Beaulieu, 63000 Clermont-Ferrand, France; 2 Syngenta, Toulouse Innovation Centre 12 Chemin de l’Hobit, 31790 Saint-Sauveur, France; 3 Cell and Molecular Sciences, The James Hutton Institute, Invergowrie, Dundee DD2 5DA, UK; 4 Institut Jean-Pierre Bourgin, INRAE, AgroParisTech, CNRS, Université Paris-Saclay, 78000 Versailles, France

**Keywords:** recombination, synapsis, wheat, chiasmata, 3D-SIM, meiosis, cytogenetics, deletion bin

## Abstract

Understanding meiotic crossover (CO) variation in crops like bread wheat (*Triticum aestivum* L.) is necessary as COs are essential to create new, original and powerful combinations of genes for traits of agronomical interest. We cytogenetically characterized a set of wheat aneuploid lines missing part or all of chromosome 3B to identify the most influential regions for chiasma formation located on this chromosome. We showed that deletion of the short arm did not change the total number of chiasmata genome-wide, whereas this latter was reduced by ~35% while deleting the long arm. Contrary to what was hypothesized in a previous study, deletion of the long arm does not disturb the initiation of the synaptonemal complex (SC) in early meiotic stages. However, progression of the SC is abnormal, and we never observed its completion when the long arm is deleted. By studying six different deletion lines (missing different parts of the long arm), we revealed that at least two genes located in both the proximal (C-3BL2-0.22) and distal (3BL7-0.63-1.00) deletion bins are involved in the control of chiasmata, each deletion reducing the number of chiasmata by ~15%. We combined sequence analyses of deletion bins with RNA-Seq data derived from meiotic tissues and identified a set of genes for which at least the homoeologous copy on chromosome 3B is expressed and which are involved in DNA processing. Among these genes, eight (CAP-E1/E2, DUO1, MLH1, MPK4, MUS81, RTEL1, SYN4, ZIP4) are known to be involved in the recombination pathway.

## 1. Introduction

Mastering genes controlling meiosis and recombination is essential for wheat breeders to better exploit the wealth of diversity that exists in wild-grass resources [1,2,3]. Meiosis is a specialized cell division that halves the chromosome complement, or ploidy, during the production of gametes in sexually reproducing eukaryotes. To ensure that each gamete has a full complement of genetic material, homologous chromosomes must pair and then separate in a coordinated manner during meiosis. With a few exceptions (e.g., *Bombix mori* in females [4], Drosophila in males [5] and *Rhynchospora tenuis* [6]), this is mediated by the formation of genetic crossovers (COs; for a review see [7]) that reshuffles genetic material between chromosomes. Chiasmata are the cytogenetic manifestation of the COs and are essential to ensure homologue alignment on the metaphase I plate. At least one CO is mandatory per homologous pair (obligate CO [7]), and the absence of COs results in the random segregation of homologous chromosomes at anaphase I. This absence of COs leads to the formation of aneuploid gametes at the end of meiosis because of the occurrence of univalent chromosomes resulting from homologous-pairing failure during the prophase I stage.

Hexaploid bread wheat (*Triticum aestivum* L.; AABBDD; 2n = 6X = 42) derives from two successive interspecific crosses [8,9,10] involving the three diploid species *T. urartu* (AA), a yet unknown species related to *Aegilops speltoides* (SS, close to BB [11,12,13]) and *Ae. tauschii* (DD). Bread wheat has, therefore, three sets (A, B, D) of highly similar (called homoeologues) chromosomes. Due to this polyploid structure, bread wheat tolerates aneuploidy quite well, probably because of the redundancy of the genetic information carried by the homoeologous chromosomes. Aneuploid lines missing a pair of one of the 21 chromosomes (nullisomic lines), a pair of chromosome arms (ditelosomic lines) or part of the chromosome (deletion lines) were produced [14,15,16,17]. They were further used for gene identification [18] as well as for molecular marker assignment [19,20]. Interestingly, the line missing chromosome 3B (nullisomic 3B) shows an aberrant meiotic behaviour [15] with numerous univalents, and it generates many aneuploid descents (monosomics or trisomics). Similarly, Naranjo [21] showed that deletions of the short arm of chromosome 3B lead to a reduction in chiasmata within this arm but do not affect genome-wide chiasmata numbers, while different deletions of the long arm of chromosome 3B result in a genome-wide reduction in chiasmata. This suggests that chromosome 3B carries essential genes required to ensure a faithful and complete meiotic recombination and that the other two chromosome-3A and -3D copies cannot compensate for the loss of 3B-homoeologous copies of these genes. However, neither of these two studies considered the complete synapsis of the aneuploids nor evidenced any candidate genes [15,21].

Chromosome 3B from bread wheat has been the most studied for more than 15 years. Due to its huge physical size, this chromosome was the first to be isolated through chromosome sorting and the first for which a BAC library was constructed [22]. Consequently, chromosome 3B was also the first for which an anchored physical map was elaborated [23] as well as a completely assembled and annotated pseudomolecule [24]. This unique and well-documented resource was further used to study the global structure, functioning and evolution of chromosome 3B [24,25,26,27]. This also provides an opportunity to identify the genes located on this chromosome that affect meiotic behaviour [15,21].

Wheat chromosomes 3A, 3B and 3D are stated as playing a role in recombination. The most well-known locus is *Ph2* (for *Pairing homoeologous 2*), a gene located on the short arm of chromosome 3D (3DS; [28,29,30]). Evidence suggests that *Ph2* acts on homologous recombination through synaptic progression [31,32], but without affecting the recombination rate in wheat [33,34,35]. Actually, *Ph2* rather affects homoeologous recombination in wheat haploids (ABD), pentaploids (AABBD) and wheat-rye allo-haploid hybrids (ABDR) [28,29]. It was recently shown that *TaMsh7*, a gene involved in mismatch repair [36,37], corresponds to *Ph2* [38]. Another gene preventing homoeologous recombination is also mentioned on 3AS, but it has a weaker effect than *Ph2* [30,39]. Its position suggests that it could be a homoeologous copy of *Ph2*. Deficiency for both 3AS and 3DS results in a level of homoeologous recombination almost as high as that expected for plants missing *Ph1* [40]. *Ph1* is a cloned locus [41] involved in homoeologous recombination and recently characterized as being a wheat-specific duplicated copy of *Zip4* (*TaZip4-B2*; [42,43]). Another unknown gene preventing homoeologous recombination and which does not interact with *Ph1* was located on the long arm of chromosome 3B [44,45,46] as well as probable homoeo-alleles on the long arms of chromosomes 3A and 3D [29,30,39].

The genomic resources available for chromosome 3B can boost cloning of the genes involved in recombination and located on chromosome 3B, as well as on chromosomes 3A and 3D, when homoeologous copies exist [18,29,30,39]. The objectives of this paper were to study the variation in chiasma number in the set of deletion lines from chromosome 3B [17] in order to locate more precisely on this chromosome the genes that are involved in this trait. Our results showed a reduction in chiasma number in the deletion lines from the long arm. The difference between the lines suggests the presence of at least two genes involved in this trait. In a second step, we used the reference sequence of chromosome 3B [47] to map in silico genes that are known to be involved in meiosis in model species in order to identify putative candidate genes implicated in recombination. We identified 10 genes affecting genome stability or cell cycle, located in the two deletion bins associated with a reduction in chiasma number. In addition, we present original results on the meiotic behaviour in the deletion line 3BL7-0.63-1.00 (hereafter named 3BL7) and in the ditelosomic line missing the long arm of chromosome 3B (Dt3BS), showing that deletion of the long arm does not prevent the initiation of the synaptonemal complex (SC) but hampers its completion.

## 2. Results

### 2.1. Chromosome 3B Is Essential for Chiasma Number but Cannot Be Compensated by Chromosomes 3A or 3D

**Figure 1:**
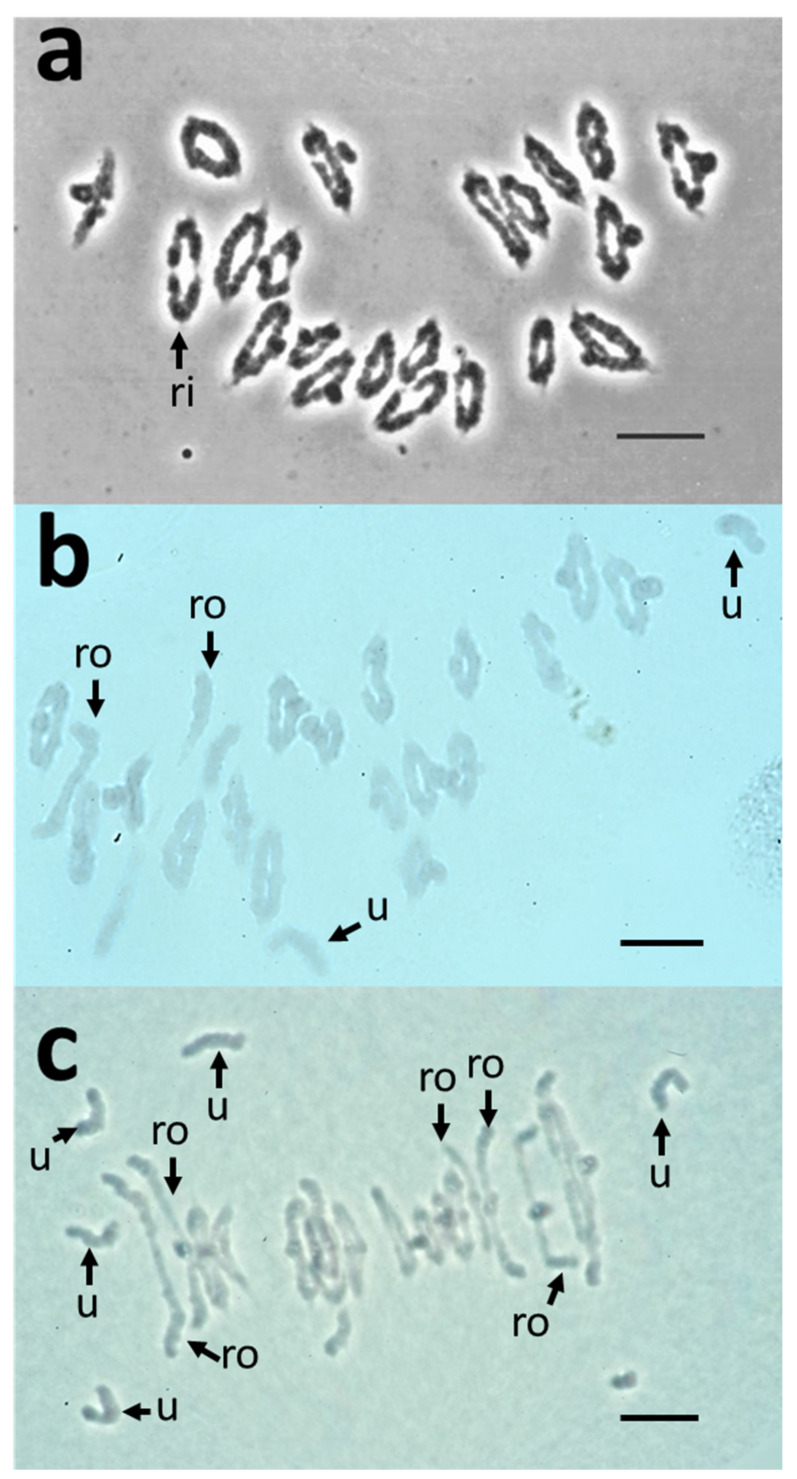
Meiotic behaviour of pollen mother cells (PMCs) of wild-type, Nulli 3B and ditelosomic Dt3BS Chinese Spring lines. Wild-type cells (**a**) exclusively show ring bivalents (ri) at metaphase I. Nullisomic 3B cells (**b**) exhibit a range of ring or rod (ro) bivalents and rare univalents (u), while ditelosomic Dt3BS cells (**c**) contain ring-bivalents and many rod bivalents (ro) and univalents (u), suggesting loss of obligate crossovers. Scale bar = 10 µm.

Numbers of bivalents (rods and rings), univalents, multivalents and chiasmata were assessed on a set of 50 meiotic (metaphase I) nuclei in all genotypes (Table 1; raw data in Table S1; statistics in Tables S2–S4). Wild type euploid Chinese Spring variety (CS; 2n = 6x = 42 chromosomes; 14 A, 14 B and 14 D chromosomes) exhibited ~21 bivalents (~20 rings and ~1 rod; Table 1; Figure 1a) and a mean of ~41 chiasmata. 

A–L: Multiple comparisons by pairs following the Dunn procedure/Bilateral test (*α* = 0.05; see Table S3 for details). Each comparison test is considered by columns only.

NA: Grouping could not be done properly as significance of differences was not transitive in this case.

As expected, almost no univalent was observed (2 in 50 PMCs), which confirmed that euploid CS exhibits a normal meiotic behaviour. On the contrary, the line missing chromosome 3B (N3B; Table 1; Figure 1b) had more univalents (mean 0.44 ± 0.84) and a significantly increased number of rod bivalents (4.34 ± 1.76; Table 1), leading to a significantly reduced number of chiasmata compared to CS (35.22 ± 1.99; Mann–Whitney *p* = 7.9 × 10^−18^; Table S4). This reduction was higher than expected (41−2 = ~39 chiasmata expected), showing that the effect of the loss of chromosome 3B was genome-wide and not limited to chromosome 3B itself. This confirmed the previous results from the literature [12,18], indicating that chromosome 3B of bread wheat is carrying essential genes involved in the control of chiasma number.

Chiasma number was also evaluated in Nulli-Tetrasomic (NT) lines (lines for which one pair of homologous chromosomes is replaced by one pair of their homoeologues) missing either chromosomes 3A, 3B or 3D. If the genes located on homoeologous group 3 chromosomes fully compensate for each other, then the same number of chiasmata is expected in all NT lines as in the euploid line (~41). Contrary to this prediction, all of the NT lines had a significantly reduced number of chiasmata compared to the euploid line (Tables S3 and S4). The aneuploid lines missing chromosome 3B (N3BT3A and N3BT3D) showed the highest difference (36.74 ± 1.98 and 37.32 ± 1.91, respectively) followed by the aneuploid lines missing chromosomes 3A (N3AT3B and N3AT3D; 38.44 ± 2.35 and 38.82 ± 2.09) and 3D (N3DT3A and N3DT3B; 39.94 ± 1.56 and 39.82 ± 1.48). This confirmed that homoeologous group 3 chromosomes bear important genes for chiasma number. This also suggests that none of the homoeologous pairs of chromosomes can fully compensate for the one missing. This finally indicates that the genes (or copies of genes) involved in chiasma number on chromosome 3B have stronger effects than those from chromosomes 3A and 3D. We thus focused our analysis on this specific chromosome.

### 2.2. The Long Arm of Chromosome 3B Is Essential for Chiasma Number in Wheat

In order to narrow down the location of this (these) gene (s), similar analyses were made on double ditelosomic lines having either two copies of the short (S; Dt3BS) or the long (L; Dt3BL) arms of chromosome 3B. Since, for these two lines, one arm is missing, we expected to observe the same number of bivalents but with an additional rod and with one ring and one chiasma missing compared to CS, i.e., ~21 bivalents, ~2 rods, ~19 rings and ~40 chiasmata. For Dt3BL, we observed ~21 bivalents (2.5 rods and 18.5 rings) and 39.44 ± 1.32 chiasmata (Table 1), which is not statistically different from expectation (Wilcoxon–Mann–Whitney; α_altered_ = 0.00185; respective *p*-values = 0.57; 0.04; 0.03; 0.03). This indicates that genes on this arm affecting chiasma number have only a limited effect or that their absence is fully compensated for by homoeologous genes on chromosomes 3A and 3D. On the contrary, when Dt3BS (Figure 1c) was analysed compared to normal CS, we observed only 17.92 ± 1.81 bivalents (10.00 ± 2.11 rods and 7.92 ± 1.82 rings) on average and 25.84 ± 2.94 chiasmata (*p* = 3.7 × 10^−18^). Especially, the number of rod bivalents was largely increased (10 times) at the expense of ring bivalents compared to normal CS. At the same time, the mean number of univalents was 6.16 ± 3.61 in Dt3BS while none were observed in euploid CS and only 0.44 ± 0.84 for N3B. These results suggest that at least one gene with a strong effect on chiasma number is present on the long arm of chromosome 3B. It is also worth noting that loss of 3BL (Dt3BS) has a stronger negative effect on chiasma frequency than loss of the entire chromosome 3B (N3B; Table 1). Since deletion of the entire short arm does not result in a decrease in chiasma number and, since deletion of the long arm only has a stronger effect than suppression of the entire chromosome, this suggests that the two arms have an opposite effect on chiasma frequencies and that the short arm of chromosome 3B may carry anti-crossover (anti-CO) genes.

We then analysed meiosis in F1 hybrids derived from the cross between CS and either Dt3BL or Dt3BS. In the first case, two doses of 3BL for one of 3BS resulted in a mean number of chiasmata (39.30 ± 1.09) that was similar (*p* = 0.4) to that observed in Dt3BL (39.44 ± 1.32). This is consistent with our hypothesis that 3BS does not carry any gene with a significant effect on chiasma number. On the contrary, one dose of 3BL for two doses of 3BS resulted in a mean frequency of chiasmata (36.44 ± 1.89), intermediate between those of Dt3BS (25.84 ± 2.94) and CS (40.98 ± 1.12). This suggests that the genes affecting chiasma number on the long arm show haplo-insufficiency (a situation in which the product of a single allele, although active, is synthesized in an insufficient quantity to allow the normal functioning of the cell).

From these results, we can conclude that (i) at least one gene located on the long arm of chromosome 3B is required to ensure WT crossover numbers; (ii) this gene shows haplo-insufficiency (i.e., is dosage-dependent) and (iii) at least another gene present on the short arm of chromosome 3B encodes an anti-crossover protein. We decided to locate this (these) gene(s) more precisely by evaluating bivalent/chiasma formation using a wider range of 3B-deletion lines from CS.

### 2.3. The Long Arm of Chromosome 3B Bears at Least Two Genes Involved in Chiasma Number

**Figure 2:**
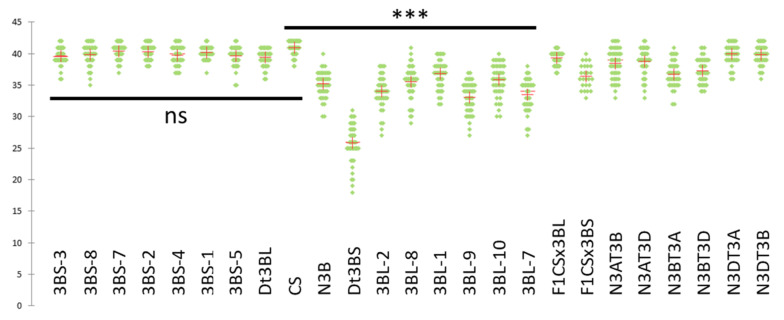
Average numbers of chiasmata (red crosses) for the various Chinese Spring lines: wild-type (CS), ditelosomic (Dt3BS and Dt3BL), deletion lines (3BS-xx or 3BL-xx for short and long arms respectively), hybrids between CS and Dt (F1xxx) and nulli-tetrasomics. ***: *p* < 0.001.

Seven deletion lines missing from 13% (3BS3) to 93% (3BS5) of the short arm of chromosome 3B were analysed. In all these lines, we observed almost no univalent (0 to 0.4/cell; Table 1; Figure S1), an average number of bivalents of ~21 and a slight variation in chiasma number between the lines, from 39.6 to 40.4 (Figure 2). These differences were not statistically significant compared to the wild type of CS (Table S3), except the number of chiasmata. However, the small drop in chiasmata and the way in which it is accentuated with larger deletions suggest an effect of the deletion in cis, i.e., deletions of the terminal part of the chromosome prevent chiasma formation in this region that is particularly prone to chiasmata in the WT. This was confirmed when the number of chiasmata of CS was reduced by one and the difference was no more significant (Table S3; 0.320 < *p* < 0.951). As we previously suggested from the Dt3BL result, this confirmed that if genes involved in chiasma formation exist on 3BS, they must have a limited effect, or their loss is fully compensated for by the presence of their homoeologues.

Similarly, six deletion lines missing from 37% (3BL7) to 78% (3BL2) of the long arm of chromosome 3B were analysed. They all highly significantly differed from CS in the number of chiasmata, that ranged from 33.10 ± 2.38 to 36.80 ± 2.16 (Table 1; Figure 2; *p* < 0.0001, Table S3). They also all significantly differed from Dt3BS (*p* < 0.05), except 3BL-9, that was just above the threshold (*α* = 0.05; *p* = 0.052). Interestingly, they did not differ significantly from N3B (0.101 < *p* < 0.543, Table S3), except 3BL-1, that was just below the threshold (*α* = 0.05; *p* = 0.039). This difference in chiasma number was explained by (1) the occurrence of more univalents that were observed in all the 3BL deletion lines ranging from 0.36 ± 0.78 to 2.20 ± 1.91/cell (Table 1 and Table 2) the increased number of rod bivalents (4.10 ± 1.69-5.80 ± 1.84/cell) at the expense of ring bivalents (13.68 ± 2.00–16.72 ± 1.73/cell). Given that the increase in univalents was paralleled by a decrease in chiasmata on bivalents, our results suggest that the deletions have an effect on genome-wide chiasma formation (in trans) in addition to an effect (in cis) at the chromosome 3B level.

The decrease in chiasma number in 3BL deletion lines and in the Dt3BS line suggests that at least two 3BL deletion bins contain genes involved in the control of this trait. Figure 2 represents the effect of these deletions on chiasma number and reveals two main drops: from ~41 to ~33 when deleting the distal bin 3BL7-0.63 and from ~33 to ~26 when deleting the proximal bin (between the centromere and 3BL2-0.22). These results suggest that the first region is located distally, in deletion bin 3BL7-0.63-1.00; this region is lost in all deletion lines, which explains why they all show less chiasmata than CS. The second region would be located proximally in deletion bin C-3BL2-0.22; this region is present in all deletion lines and only lost in Dt3BS, which explains why this line showed less chiasmata than any deletion lines. From the results of the analysis of the chromosome 3B deletion lines, we can conclude that: (1) no gene with a significant effect regarding chiasma reduction is located on the short arm; (2) the long arm of chromosome 3B carries at least two genes involved in chiasma number.

### 2.4. Comparative Analysis of Meiotic Behaviour in CS, 3bl7 and dt3bs Lines

**Figure 3:**
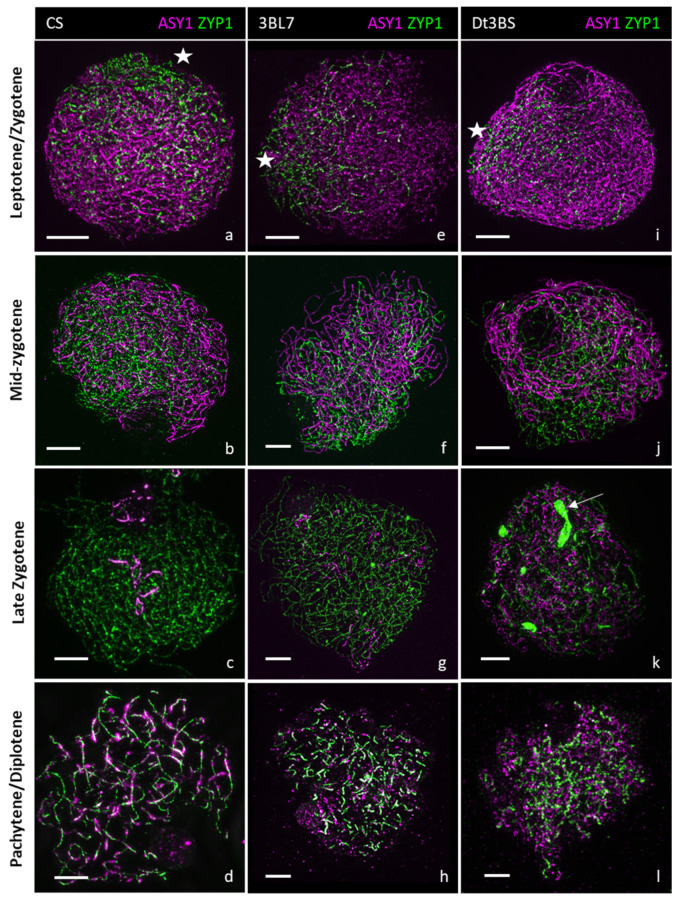
Comparison of synapsis in CS, 3BL7 and Dt3BS by 3D super resolution imaging. Chromosomes axes are labelled with ASY1 (magenta), and ZYP1 (green) is used to follow synapsis. In CS (**a**–**d**), synapsis starts at one side of the nucleus (white star; **a**) and progressively brings the homologues together at zygotene (**b**,**c**). Chromosomes are fully synapsed at the pachytene/diplotene stage (**d**). In 3BL7 (**e**–**h**), synapsis resembled that of the wild type of CS. In Dt3BS, the initiation of synapsis (**i**) and the progression at zygotene (**j**) are similar to CS. However, during zygotene, ZYP1 forms polycomplexes (white arrow; **k**) during synapsis, suggesting a difficulty to polymerize the central element of the synaptonemal complex, although chromosomes seem aligned with a dotty synapsis at pachytene (**l**). Scale bar = 5 µm.

We analysed the meiotic behaviour of lines Chinese Spring (CS; control), Dt3BS and 3BL-7. Using antibodies raised against HvZYP1 [48] and the axial element associated protein TaASY1 [49], combined with Structured Illumination Microscopy (SIM), we compared synapsis in CS, Dt3BS and 3BL7 (Figure 3). Axis formation, tracked by TaASY1 (magenta), appeared normal in all three lines, as shown by the regular ASY1 signal. In CS, at the onset of the zygotene stage, the synaptonemal complex (visualized with HvZYP1 antibody) starts to polymerize at one side of the nucleus in CS (Figure 3a), presumably at the telomere region [50,51], and brings the chromosomes in a zipper-like manner (Figure 3b,c). At the pachytene stage, the chromosomes aligned along each other via the synaptonemal complex (Figure 3d). The line 3BL-7 displays normal synapsis from zygotene to pachytene stages (Figure 3e–h), which is not significantly different from the wild type. In the Dt3BS line, synapsis appears similar to CS at the leptotene/zygotene stage (Figure 3i) and at the mid-zygotene stage (Figure 3j). However, by the late zygotene stage (Figure 3k), Dt3BS exhibits ZYP1 aggregates, which resemble a polycomplex, suggesting a defect in the maturation of the synaptonemal complex (ZYP1-polycomplex means that the SC is not normal, but that the ZYP1 protein is still in the nucleus and forms a polymer; [52]). At the pachytene stage (Figure 3l), although the chromosomes are fully aligned, ZYP1 is not fully polymerized, revealing an abnormal synapsis.

Thus, contrary to what was hypothesized in a previous study, Dt3BS is not fully asynaptic, since we observed the formation of the synaptonemal complex (SC) in the earlier stages. However, progression of the SC is abnormal in this line, and we never found a complete synapsis.

**Figure 4:**
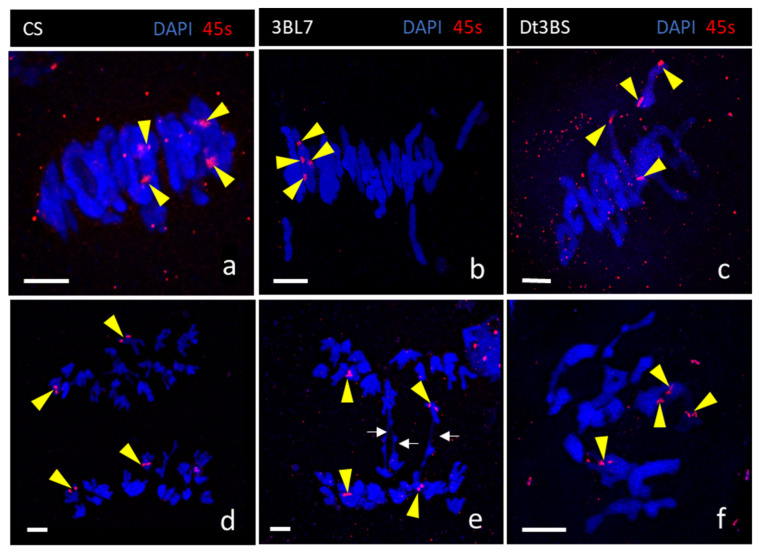
Homologous chromosome pairing and segregation at Metaphase I and Anaphase I in lines CS (**a,d**), 3BL7 (**b,e**) and Dt3BS (**c,f**). Homologous pairing was monitored using the 45S-rDNA probe. All lines exhibit correct homologous chromosome pairing as shown by the localisation of two 45s signal (red, yellow arrows) per bivalent (DAPI blue). Homologous chromosomes halve at anaphase I in both CS (**d**) and 3BL7 (**e**), with occasional chromosome bridges (white arrows) in 3BL7. We could not find a similar stage for Dt3BS. Moreover, Dt3BS chromosomes were “fuzzier” and stickier at Metaphase I (**f**) than in the other lines, making chromosome spread for 3D DNA in situ hybridization in this line more challenging. Scale bar 10 µm.

We also checked for possible non-homologous chromosome pairing using fluorescent DNA in situ hybridization of the rDNA probe 45S [53,54] in the same three lines, CS, 3BL-7 and Dt3BS (Figure 4). We detected the four largest 45S rDNA sites, two per homologous bivalents, on every metaphase. In CS (Figure 4a), we detected two ring bivalents carrying the two 45S signal at the telomere region. In 3BL-7 line (Figure 4b), we also detected bivalents (ring or rod) carrying two signals for 45S, suggesting that, in the 3BL7 line, homologous chromosome pairing is not affected. Similarly, the Dt3BS line (Figure 4c) seems to have normal homologous chromosome associations. Chromosome segregation occurred normally in CS (Figure 4d) and 3BL7 (Figure 4e), as shown by the same number of 45S-rDNA (red) signals equally segregating. Occasional anaphase bridges were visible, even in CS (wild type). We were not able to find the corresponding anaphase for Dt3BS, as meiotic staging is difficult in this line. However, in Dt3BS, the chromosomes appeared “fuzzier”, suggesting difficulties in chromatin compaction/condensation (Figure 4f). Altogether, our results suggest that chromosome recognition is not affected in Dt3BS and 3BL-7 and that the reduction in chiasmata (crossover) is likely due to a defect in the crossover pathway during meiosis.

### 2.5. In Silico Mapping of Genes Expressed during Wheat Meiosis or Known as Involved in Meiosis in Arabidopsis

We used the pseudomolecule of chromosome 3B [47] and our RNA-Seq data from wheat developing anthers [55] to identify genes expressed during meiosis and located within the different deletion bins (Table 2). Among the 6060 high-confidence (HC) or manually annotated (MA) genes located in our deletion bins on chromosome 3B (Table S5), 3805 and 2255 were located on the long arm and the short arm, respectively. RNA-Seq data revealed 2099 genes (mean 34.64%; 33.08% and 35.56% for short and long arm, respectively) expressed during meiosis (FPKM > 5) and physically assigned to deletion bins (Table 2). The number of expressed genes in the different deletion bins varied greatly and ranged from 18 (3BS7-0.75-0.78) to 583 (3BL7-0.63-1.00), reflecting the difference in bin size ranging from 4.5 Mb (3BS3-0.87-1.00) to 205 Mb (3BL7-0.63-1.00).

Based on gene ontology (http://www.informatics.jax.org/vocab/gene_ontology/ (accessed on 23 August 2022); 5578 genes, 36.7%) or on their putative function (Table S6), at least 70 HC genes (Table S5; 32 on 3BS and 38 on 3BL) could play a role in the meiosis pathway. For example, two of them are good candidates that may explain the meiotic behaviour we observed when the short arm is absent. TraesCS3B02G048300 (3BS8-0.78-0.87) is annotated as a member of the Fanconi anemia group M protein, related to FANCM, that acts as an anti-crossover protein [56]. TraesCS3B02G136600 (3BS4-0.55-0.57) is annotated as a DNA mismatch repair from the MutS family, that could also restrain crossover formation between non-identical chromosomes [38,57,58].

To confirm this, we selected a set of 104 genes from Arabidopsis, known to be involved in meiosis or playing a role in DNA repair (Supplementary Table S7), to identify orthologous genes on 3B. Five mapped on the short arm of chromosome 3B (Table 3; BRCA2, MSH7, DUO1-3S, CYCA1;2/TAM1 and MUS81-3S), but none of them were known as being involved in the anti-CO pathway. Eleven of them mapped on the long arm (Table 3), among which five were located in bin C-3BL2-0.22 (RTEL1, DUO1-3L, and three copies of MPK4) and five in bin 3BL7-0.63-1.00 (CAP-E1/E2, SYN4, ZIP4, MUS81-3L and MLH1).

**Figure 5:**
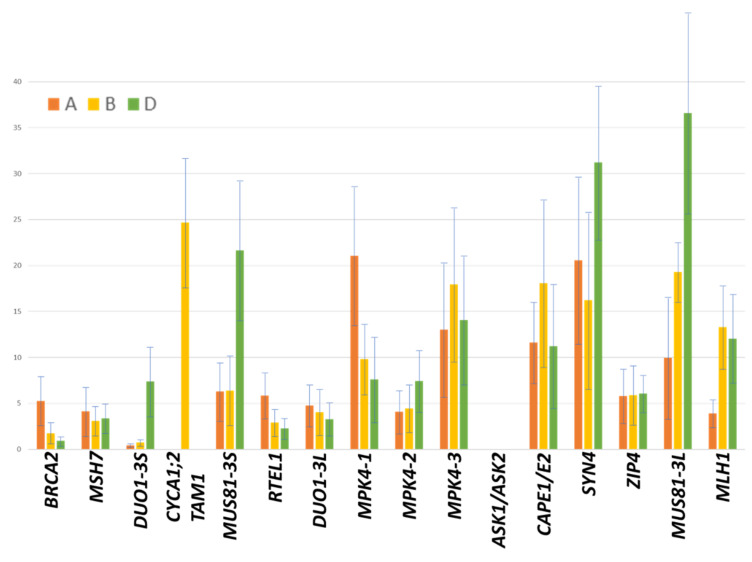
Level of expression (FPKM values; average of the four stages and two replicates) in meiotic tissues [55] of the different homoeologous copies (**A**: orange; **B**: yellow; **D**: green) of genes known as being involved in recombination in model species and located on chromosome 3B.

Interestingly, DUO1 and MUS81 have duplicates on both arms of this chromosome and MPK4 has triplicates in the same deletion bin. We compared the expression of these candidate genes with that of their homoeologous copies using a set of already available RNA-Seq data from the literature as transcript reference [55]. Copies from chromosome 3B were the most highly expressed for CAP-E1/E2, MLH1 and MPK4-3 (Figure 5). For ZIP4, the expression level of the three copies was balanced. These genes can be considered as good candidates responsible for the control of chiasma formation.

We evaluated the potential functionality of the three copies for all these genes by comparing the protein sequence of eleven wheat varieties with those from the diploid species (*T. urartu* and *Ae. tauschii*) that should be functional to ensure a good fertility of these two species (Table S8). Based on these analyses, we estimate that all A and D copies of the 16 genes should be functional except BRCA2-A, MPK4-D1, MPK4-A3 and ZIP4-A. We then compared the A, B and D copies, and results suggest that MSH7-3B, RTEL-3B and MUS81-3BL may not be functional. However, this must be confirmed using a genetic approach with appropriate mutants.

## 3. Discussion

### 3.1. Variation in Chiasma Number in the Aneuploid Stock from Chromosome 3B

Chromosome 3B has been found to carry genes playing a role in pairing in many studies [18,21,44,45,46,59]. In this study, we used the aneuploid stocks of wheat, namely, the nullisomic, nulli-tetrasomic, ditelosomic and deletion lines related to chromosome 3B, to locate the genes involved in recombination and chiasma number on this chromosome. Due to the polyploid nature of the wheat genome, homoeologous copies can compensate for the lack of one copy. Thus, only genes that are present in a single copy, those that are sensitive to a dosage effect or those with the 3B copy having the major effect, could be detected in our study. Moreover, our observations rely on chiasma counting of Pollen Mother Cells (PMC) that underwent a complete meiosis until metaphase I. However, the genes concerned by the deletions could have affected the earlier phases of meiocyte development and, perhaps, a large number of cells could have died before reaching metaphase. We observed a fair amount of synapsis even in Dt3BS, although we could never find a complete synapsis in this line. The timing of meiosis may be disturbed, and we saw cells that could have been late at synapsis. A time course analysis would allow us to answer this question. We did not observe loss in fertility in any of the aneuploid lines we used. This would have been important information, since mutated genes could reach an earlier control for pairing and recombination with regard to the observation of meiotic behaviour.

We observed a significant but limited effect of the partial or total loss of the short arm of the chromosome 3B on chiasma number. This suggests that this chromosome arm carries genes involved in this trait, but with only a tiny effect. Interestingly, deletion of the long arm only (Dt3BS) has a stronger effect than suppression of the entire chromosome (N3B) with~26 and ~35 chiasmata, respectively. This suggests that the two arms have an opposite effect on chiasma frequencies and that anti-crossover genes locate on the short arm of chromosome 3B. Anti-crossover protein-encoding genes have been found in Arabidopsis, such as FANCM, FIGL1, RECQ4 and TOP3α [56,60,61,62,63,64,65]. None of the true wheat orthologues of these genes are located on the short arm of homoeologous group 3 chromosomes. However, we found high-confidence genes annotated as members of the FANCM and TOP3α proteins. There are also several other DNA helicase-encoding genes that have been predicted. This suggests that if anti-crossover protein-encoding genes are present on the short arm of chromosome 3B, they are different from those that have already been isolated. This may also reflect the fact that some genes from the short arm of chromosome 3B act as a complex with those from the long arm and/or regulate their activity. Once the short arm only is present, the genes it carries may more strongly regulate the homoeologous copies from chromosome arms 3AL and 3DL.

On the contrary, partial or total deletion of the long arm of chromosome 3B revealed more contrasted results. Deletion of the terminal bin (205 Mb) strongly disturbs the number of chiasmata. In addition, complete removal of the long arm increases this perturbation of pairing and decreases recombination. This suggests the presence of at least two genes, one located in deletion bin 3BL7-0.63-1.00 and the second in C-3BL2-0.22. Naranjo [21] also observed a reduced number of chiasmata in deletion lines 3BL6-0.54 and 3BL3-0.41 (23.3 and 29.6, respectively), while the deletion line 3BL11-0.81 exhibited normal behaviour (40.2 chiasmata). This allows for refining the position of the gene located distally in the interval determined by the breakpoints from the deletion lines 3BL7-0.63 and 3BL11-0.81, which halves the size of the segment from 205 Mb to 100 Mb.

The presence of a pairing promoter gene located on the long arm of chromosome 3B is necessary for normal synapsis and chiasma formation [18,59]. This gene is probably the one we identified in bin 3BL7-0.63-1.00. Evaluation of wheat radiation hybrids indicated the presence of a gene (*TaDES2*) which, when deleted, leads to a reduced fertility that could be due to a problem in pairing [66], which would confirm our results. In addition, Dt3BS was previously described as asynaptic, which implies a defect in pairing [67]. Implication of the long arm of chromosome 3B in pairing is in accordance with previous results which mentioned the effect of this region, either directly in wheat [18,35,46] or with crosses with related species such as *Aegilops* [68,69]. Moreover, the homoeologous chromosome arms, 3AL and 3DL, carry promoters of pairing that could be homoeo-alleles of the gene on 3BL [28,29,39].

### 3.2. Identification of Candidate Genes Located in the Two Deletion Bins Concerned

Combining in silico assignment of genes known to be involved in meiosis and RNA-Seq data led to the identification of several candidate genes in deletion bins C-3BL2-0.22 (RTEL1, DUO1 and MPK4) and 3BL7-0.63-1.00 (MLH1, ZIP4, SYN4, CAP-E1/E2 and MUS81), among which the 3B copies of MPK4, MLH1 and CAP-E1/E2 were probably functional and more expressed than their homoeologues.

MPK4 (Mitogen-activated protein kinase) was initially described as playing a role in regulating cortical microtubule bundling [70], but, recently, it was evidenced that *mpk4* mutants develop normal microspore mother cells that further fail to complete meiotic cytokinesis, leading to aborted pollen grains [71]. Here, we find a related MPK4 protein expressed during meiosis, suggesting an implication in regulating meiosis division. The meiotic phenotype we observed does not suggest that cytokinesis are affected in our deletion lines. Thus, MPK4 would not be the best candidate, even if we could not exclude that it can be involved in the regulation of another gene that, if less or differentially expressed, will lead to the appropriate phenotype.

MLH1 belongs to the DNA mismatch repair system (MMR) and is the homologue to the *MutL* gene originally described in *Escherichia coli*. Three homologues of *MutL* exist in Arabidopsis (*AtMLH1*, *AtMLH3* and *AtPMS1*; [72,73]). It was shown that *AtMLH1* and *AtMLH3* act as dimers during the pachytene stage of meiosis [74]. The mutation of *AtMLH1* evidenced that it is required for homologous recombination and that it also acts to limit recombination between divergent sequences [75]. In bread wheat, it was shown that the *Ph1* locus [40] prevents MLH1 sites from becoming crossovers on paired homoeologues during meiosis [76]. In our study, MLH1 could be a good candidate to explain the reduction in chiasma number since it contributes to resolving the crossovers at the end of meiosis. If MLH1 is inactivated, we should thus observe less chiasmata compared to the wild type.

*CAP-E1* is a functional orthologue of *SMC2* (Structural Maintenance of Chromosomes 2), a gene that belongs to the condensin complex involved in mitotic chromosome condensation and dosage compensation in yeast [77]. Two copies (*CAP-E1* and *CAP-E2*) were identified in Arabidopsis, and mutant analysis revealed that the genes are expressed during meiosis and that double heterozygous mutants exhibited a reduction in condensation at metaphase and anaphase I and a mild cut-type phenotype at anaphase I [78]. Since chiasma resolution requires that sister chromatids decatenate from each other along the chromosome length, when *CAP-E1* and/or *CAP-E2* are mutated, this decatenation fails, and the failure results in a lower number of chiasmata and a higher number of univalents. Moreover, chiasma frequency is significantly reduced from 9.05 chiasma per cell in the wild type to 8.15–8.44 chiasma per cell in *AtSMC4* RNAi mutants [79], suggesting a role of the condensin complex during meiotic prophase I. *CAP-E1* could thus be a good candidate as well to explain our reduced number of chiasmata in our deletion lines.

Despite the fact that the homoeologous copy from chromosome 3B of these three genes is the most expressed among the three copies, and that they thus constitute preferential candidates, we cannot exclude that, when they are deleted, the expression of their 3A and/or 3D homoeologous copies could be upregulated to compensate for their absence. This would lead to an absence of any meiotic phenotype. Looking at the expression of these genes in different deletion stocks such as N3B, Dt3BL or Dt3BS could enable us to dissect whether their expression level may indeed play a role in the observed phenotype.

We cannot exclude that the other genes that we located in the two deletion bins also play a role in the phenotype observed. For example, ZIP4 is involved in the formation of the synaptonemal complex in rice [80] but not in Arabidopsis [81] and SYN4 in centromere cohesion [82]. MUS81 is involved in class II crossovers and resolves aberrant intermediates induced during replication as well as for efficient synthesis-dependent strand annealing (SDSA) and, to a smaller extent, for single-strand annealing (SSA) [83]. It could thus affect homologous recombination frequency, and its removal would explain the reduced number of chiasmata that we observed in the lines missing part or all of the long arm of chromosome 3B.

Moreover, we had more than 800 genes that were expressed during meiosis in the 2 deletion bins C-3BL2-0.22 and 3BL7-0.63-1.00. We cannot exclude that at least one of these genes that is currently unknown as playing a role in meiosis could be responsible for the observed phenotype. Then, mutants for these genes will have to be analysed individually, and the resources developed for mutant identification (http://www.wheat-tilling.com/, accessed on 20 April 2022) will be of main importance to accelerate this process. Finally, all the genes that are located in the two bins may also act in complexes or may regulate each other, which will largely complicate the identification of those having the largest impact on the phenotype we observed.

### 3.3. Conclusions

Our study showed that at least two genes impact chiasma number and locate distally (3BL7-0.63-1.00) and proximally (C-3BL2-0.22) on the long arm of chromosome 3B of bread wheat. The formation of bivalents at meiosis results from three major processes: chromosome pairing itself, which is the interaction between homologous chromosomes resulting in their alignment; synapsis, which is the formation of the synaptonemal complex between homologues; and crossovers, which occur at the end of prophase I. Meiotic behaviour of our mutants needs to be better deciphered to understand which part of the process is impacted and to better focus on the appropriate candidates. The recent genomic resources (whole-genome sequence [46], expression data [84]) that are now available for wheat provided us with the opportunity to find putative candidate genes. They now need to be studied in more detail by searching for mutants in TILLING populations (http://www.wheat-tilling.com/, accessed on 20 April 2022) or by developing transgenic plants (knock-out or over-expression) and evaluating the chiasma number and recombination behaviour of the mutants.

## 4. Materials and Methods

### 4.1. Plant Material

To estimate the location of genes located on chromosome 3B involved in chiasma number at meiosis, we used the reference line Chinese Spring as a control and the aneuploid stock of chromosome 3B described earlier [16,17,18,85]. The aneuploid lines consisted of the nullisomic 3B (N3B), the nullisomic–tetrasomic (NT) lines, two ditelocentric lines (Dt3BL and Dt3BS) and thirteen chromosome-3B deletion lines (Table 1), seven for the short arm (3BS3-0.87, 3BS8-0.78, 3BS7-0.75, 3BS2-0.57, 3BS4-0.55, 3BS1-0.33, 3BS5-0.07) and six for the long arm (3BL2-0.22, 3BL8-0.28, 3BL1-0.31, 3BL9-0.38, 3BL10-0.50, 3BL7-0.63). Each deletion line is defined by the fraction length of the arm that remains present on the deleted chromosome (for example, line 3BS4-0.55 conserves proximal 55% and lacks the distal 45% of the short arm of chromosome 3B). These deletion lines together with Dt3BL and Dt3BS define 15 deletion bins: 3BS3-0.87-1.00, 3BS8-0.78-0.87, 3BS7-0.75-0.78, 3BS2-0.57-0.75, 3BS4-0.55-0.57, 3BS1-0.33-0.55, 3BS5-0.07-0.33, C-3BS5-0.07, C-3BL2-0.22, 3BL2-0.22-0.28, 3BL8-0.28-0.31, 3BL1-0.31-0.38, 3BL9-0.38-0.50, 3BL10-0.50-0.63 and 3BL7-0.63-1.00. Each deletion bin is defined by the fraction length of two successive deletion lines (for example, the deletion bin 3BS4-0.55-0.57 is the chromosome fragment covering the region between 55% and 57% of the short arm of chromosome 3B and delimited by deletion lines 3BS4-0.55 and 3BS2-0.57). Plants grew in growth chambers until the first two leaves emerged. They were then potted and grown for two weeks in cool conditions (17 °C day and 12 °C night, 12 h of day) and then for two months at 24 °C during the day and 18 °C during the night with 16h of day until the spikes arrive at the appropriate stage to observe chiasmata (metaphase I). Two plants for each studied line were sown to check for the homogeneity of the deletion and one of the two plants was randomly selected for the cytogenetic analyses.

### 4.2. Chiasma Observation and Statistical Analysis

Young spikes were collected and the metaphase I stage was determined by evaluating one anther per flower through a rapid squash under carmine–acetic coloration. The two other anthers were fixed in a solution of alcohol/acetic acid (3/1) for 48 h and were then dissected in aceto-carmine added with FeCl_3_ using an Axioskop (Zeiss) dissecting microscope. Only pollen mother cells (PMCs) were conserved for further analyses. The slides were heated until separation of the chromosomes and were washed with acetic water (45%). They were then slightly pressed to spread out the chromosomes and they could be stored in the fridge for rapid observation or in alcohol at −20 °C after removal of the slide. Cytoplasmic proteins were removed with pepsin followed with a paraformaldehyde treatment and the slides were washed twice (2X SSC) and fixed in successive alcohol solutions (70%, 95% and 100%). Squashed anther preparations were then viewed using the Axioskop. To estimate chiasma frequencies, 50 PMCs in metaphase I were scored for each plant and numbers of univalents, rod and ring bivalents and multivalents were recorded (Table S1). To estimate the mean chiasma frequencies, we assumed that univalents, rod bivalents, ring bivalents, trivalents and quadrivalents contribute respectively with zero, one, two, three and four chiasmata.

Normality of the data was assessed using a Shapiro–Wilk test (Table S2). Significant differences between mutant and corresponding wild-type control chiasma frequencies were computed using either non-parametric Kruskal–Wallis (Table S3) or Mann–Whitney (Table S4) tests adjusted (Bonferroni correction) for multiple comparisons.

### 4.3. Rna-Seq Data Production and In Silico Gene Mapping

To see if the genes that we located in the relevant deletion bins were indeed expressed, and if we could find any difference between gene expression of each of the homoeologous copies, we used already available RNA-Seq data from the literature as a transcript reference [55]. These latter were derived from four different stages of developing anthers (Latent/Leptotene; Zygotene/Pachytene; Diplotene/Diakinesis; Metaphase I; data publicly available: http://wheat-urgi.versailles.inra.fr/Seq-Repository/Expression; accessed on 20 December 2021). Briefly, total RNAs were extracted from 30 to 50 anthers (depending on the stage) of the hexaploid wheat cv Chinese Spring and from 10 mg of anthers using the Macherey-Nagel Nucleospin RNA-XS kit, according to the manufacturer’s instructions. RNA-Seq non-oriented libraries were constructed in duplicates using the TruSeq kit (Illumina). The eight libraries were sequenced (GATC, Konstanz, Germany) on two lanes (four samples per lane) of HiSeq2000 (Illumina) with paired-end sequence (500 bp) in 2 × 100 bp, which generated 40 to 50 million pairs of reads per sample. For alignment of the reads, we used the sequences available through the International Wheat Genome Sequencing Consortium (http://www.wheatgenome.org/, accessed on 20 April 2022). All the reads from the RNA-Seq libraries were mapped on the scaffolds representing the gene models produced from the assembly of the reads [47]. We used TopHat2 v2.0.8 (http://tophat.cbcb.umd.edu/; [86]) with the default parameters, except that we tolerated no mismatches or splice-mismatches. PCR duplicates that are generated during library construction were removed from the analysis using SAMTOOLS (rmdup option). The transcripts reconstruction and expression determination (FPKM; [87]) were analysed with Cufflinks v2.0.2 (http://cufflinks.cbcb.umd.edu/). The genes were previously annotated, and mapping of the reads was performed on exons only, which allowed discrimination of expression of the homoeologous copies.

For candidate-gene identification, we selected the genes expressed during meiosis and a set of 104 genes (see Supplementary Table S7) known to be involved in meiosis in Arabidopsis, rice, maize and *Aegilops*. We mapped all these genes onto the pseudomolecule of chromosome 3B [47,88] using their protein sequence determined through an exonerate (V2.2.0) analysis [89]. We confirmed their position using RNA-Seq data. Only those showing the best hits for alignment (scores > 500, identity > 40%, similarity > 60%) were considered as relevant. Only predictions with an FPKM value > 5 in at least one condition were considered for expression analysis. In addition, a comparative analysis of expression between homoeologous copies was conducted. Expression on homoeologous chromosomes 3A and 3D of genes identified on chromosome 3B was searched for using the IWGSC survey protein database and blastp. Homoeologous gene copies were selected according to the closest similarity with the copy from chromosome 3B.

For comparison of the functionality of the different copies (Table S8), we used available sequences from the diploid species *T. urartu* (AA), *Ae. tauschii* (DD), the tetraploid species *T. durum* (cv. Svevo; AABB), *T. diccocoides* (cv. Zavitan; AABB) and 11 hexaploid wheat varieties (AABBDD; CS, Arina-Lr-Forno, Jagger, Julius, Lancer, Landmark, Mace, Norin-61, Spelt, Stanley, SY-Mattis [90]). Various copies were retrieved through a blasp-p analysis [88] using the sequence from the corresponding proteins in Arabidopsis (Table S7). The different copies were then aligned to identify differences. We hypothesize that the copies are functional in the diploid progenitors. First, the A and D copies of *T. urartu* and *Ae. tauschii* were compared to identify neutral mutations that do not affect functionality. These latter were removed from further analyses. Then, all A and D copies from the tetraploid and hexaploid varieties were compared separately to the copies of *T. urartu* and *Ae. tauschii*, respectively, to identify potential recurrent deleterious mutations in the A or D copies that may suggest loss of functionality in either of the two. Finally, the B copies of the tetraploid and hexaploid varieties were compared to their homoeologous A and D copies to check for their putative functionality.

### 4.4. Immunostaining

Samples were prepared according to Colas et al. [91] with slight modifications for wheat [48]. Four rounds (sown at two weeks intervals in the same growing condition) of three plants per pot for each genotype were used. Three to four tillers of each plant (excluding the main tiller) were examined, and each floret was staged under acetocarmine before proceeding to the cytological experiment. Since it is not possible to find all the different stages in only one plant, the experiments were conducted over several weeks. In wheat, each floret corresponds to a different meiotic stage along the spike, but all three anthers per floret were synchronized [92]. For each line, we selected anthers of the same size, and we observed them to evaluate the meiotic stage before proceeding to the next steps. Each experiment was conducted in both wild type and deletion lines as described in [48]. For each line, between 10 and 30 cells were observed.

Staged anthers were collected in 1X-PBS containing 0.5% Triton® X100 in an embryo dish and subsequently fixed in freshly made 4% formaldehyde solution for 30 min at room temperature. Fixed anthers were washed twice in 1X-PBS/0.5% Triton® X100 and tapped to release the meiocytes in the embryo dish. Meiocytes (30 µL) were transferred onto a Polysine® slide (Poly-L-Lysine coated slides) and left to air-dry gently. Slides were first incubated for 1h at room temperature in 1X-PBS, 1% Triton® X100 for 30–45 min to permeabilize the cells in a blocking solution consisting of 3% BSA in 1X-PBS, 0.1% TritonX100. Slides were incubated in the primary antibody solution in a wet chamber for 1h at room temperature followed by 24 to 36 h in the fridge (4–6 °C). The primary antibody solution consisted of one anti-TaASY1 (rabbit, 1:5000) [49,93] and anti-HvZYP1 (rat, 1:500) [48]. After incubation, slides were let for an additional 1h at room temperature before washing them for 15 min in 1X-PBS and incubated for 90–120 min at room temperature in the secondary antibody solution consisting of a mixture of anti-rabbit Alexa Fluor (488 or 568) and/or anti-rat Alexa Fluor (568 or 488) diluted in 1X-PBS (1:300). After a 15min wash in 1X-PBS, the slides were counterstained with 1 μg/mL Hoescht 33342 for 10–15 min, mounted in Vectashield (H-1000) and sealed with nail varnish.

### 4.5. Fluorescence DNA In Situ Hybridization

The protocol is adapted from Colas et al. [91]. Briefly, anthers were fixed in Ethanol:Acetic Acid (3:1) for at least 24 h and then transferred into 70% ethanol for storage. Anthers were spread on polysine slides, according to [51], and slides were air-dried. The 45S probe was prepared from the 45S rDNA clone from wheat (pTa71 [94]) and labelled with biotin, according to [95,96]. The hybridization mix (50% deionised formamide, 20% dextran sulfate, 1X PIPES/EDTA buffer (100:10), 0.3 M NaCl, 500 ng of salmon sperm blocking DNA, and 50 ng of each probe) was denatured for 8 min at 100 °C and immediately placed on ice to cool down for 5 min. Slides were successively dehydrated in 30, 50, 70 and 100% ethanol. Ten µL of the probe mixture was applied onto the tissue, covered with a plastic coverslip and denatured on a hotplate for 5 min at 75 °C. Slides were moved into a wet chamber and incubated at 37 °C overnight. The next day, slides were washed at 42 °C in 20% formamide, 0.1X SSC for 10 min, then in 2X SSC for 10 min. This was followed by a wash at room temperature in 2X SSC for 10 min, and in 4X SCC, 0.2% Tween 20 for 10 min. A blocking solution, consisting of 5% BSA in 4X SSC, 0.2% Tween 20 was applied for 5 min in a humidity chamber at room temperature. Biotin-labelled probes were detected with extravidin-cy3. Slides were counter stained in 1μg/mL Hoescht 33342.

### 4.6. Microscopy and Imaging

The 3D-SIM acquisition of individual meiocytes was sequentially obtained using an OMX Blaze structured illumination microscope (GE Applied Precision) for three channels (405, 488 and 568 nm); SIM image reconstruction and processing were performed with the commercial software Softworx (GE Applied Precision) [97]. The 3D Confocal stack images were acquired with LSM-Zeiss 710 for the three channels sequentially, with an averaging of at least four, and de-convolved with the Tikhonov–Miller algorithm from the DeconvolutionLab package [98] for Fiji/ImageJ [99]. Projections of 3D pictures and light brightness/contrast adjustment were performed with the public domain program Fiji and/or Imaris 7.1 (Bitplane).

## Figures and Tables

**Table 1:** Number of chiasmata in the different aneuploid lines. Average numbers of chiasmata, univalent, bivalent (rods and rings) and multivalent chromosomes for the wild-type Chinese Spring line (CS) and for the aneuploid lines (nullisomic-3B (N3B); nullisomic-tetrasomic-(NxxTxx); ditelosomic Dt3BL and Dt3BS; hybrids CS x ditelosomic (F1CSx3BS and F1CSx3BL); deletion lines (3BSxx or 3BLxx). % arm: percentage of the chromosome 3B or chromosome arm present (L: Long arm; S: Short arm).

Line	% Arm	2n	Chiasmata	Univalents	Bivalents	Rods	Rings	Multivalents
CS	100	42	40.98 ± 1.12 A,B	0.04 ± 0.28 E	20.98 ± 0.14 B	1.02 ± 1.12 NA	19.98 ± 1.12 A,B	0 C
N3B	0	40	35.22 ± 1.99 H,I,J	0.44 ± 0.84 D,E	19.78 ± 0.42 J,K,L	4.34 ± 1.76 B,C	15.44 ± 1.83 F,G,H	0 C
N3AT3B	100	42	38.44 ± 2.35 D,E,F,G	0.04 ± 0.28 E	20.42 ± 0.91 B,C,D,E,F	2.34 ± 1.49 D	18.08 ± 1.69 C,D,E	0.28 ± 0.45 B
N3AT3D	100	42	38.82 ± 2.09 C,D,E,F,G	0.28 ± 0.70 D,E	20.22 ± 0.95 D,E,F,G,H,I,J	2.90 ± 1.93 C,D	17.32 ± 1.96 D,E,F	0.32 ± 0.47 A,B
N3BT3A	0	42	36.74 ± 1.98 G,H,I,J	0.88 ± 1.14 C,D	19.46 ± 1.16 I,J,K,L	4.34 ± 1.49 B,C	15.12 ± 1.75 G,H	0.56 ± 0.50 A
N3BT3D	0	42	37.32 ± 1.91 F,G,H,I	0.52 ± 0.89 D,E	19.70 ± 0.91 G,H,I,J,K,L	4.08 ± 1.91 B,C,D	15.62 ± 2.18 F,G,H	0.52 ± 0.50 A,B
N3DT3A	100	42	39.94 ± 1.56 B,C,D	0.08 ± 0.40 E	20.28 ± 1.07 B,C,D,E,F,G,H	1.92 ± 1.56 NA	18.36 ± 1.75 C,D,E	0.34 ± 0.52 A,B
N3DT3B	100	42	39.82 ± 1.48 B,C,D	0.08 ± 0.40 E	20.24 ± 0.96 C,D,E,F,G,H,I	1.98 ± 1.38 NA	18.26 ± 1.58 C,D,E	0.36 ± 0.48 A,B
Dt3BL	L	40+2t	39.44 ± 1.32 B,C,D	0.08 ± 0.40 E	20.96 ± 0.20 B	2.48 ± 1.23 D	18.48 ± 1.27 B,C,D,E	0 C
Dt3BS	S	40+2t	25.84 ± 2.94 K	6.16 ± 3.61 A	17.92 ± 1.81 L	10.00 ± 2.11 A	7.92 ± 1.82 I	0 C
F1CSx3BS	100	41+t	36.44 ± 1.89 G,H,I,J	2.00 ± 1.47 A,B	19.84 ± 0.99 F,G,H,I,J,K,L	4.16 ± 1.77 B,C,D	15.68 ± 1.89 F,G,H	0.44 ± 0.51 A,B
F1CSx3BL	100	41+t	39.30 ± 1.09 C,D,E,F	0.16 ± 0.55 D,E	20.92 ± 0.27 B,C,D	2.54 ± 1.11 D	18.38 ± 1.07 C,D,E	0 C
3BS3	87	42	39.64 ± 1.32 B,C,D	0.04 ± 0.28 E	20.98 ± 0.14 B	2.32 ± 1.33 D	18.66 ± 1.32 B,C,D	0 C
3BS8	78	42	39.92 ± 1.63 B,C,D	0.12 ± 0.48 E	20.94 ± 0.24 B,C	1.96 ± 1.58 NA	18.98 ± 1.58 B,C,D	0 C
3BS7	75	42	40.40 ± 1.21 A,B,C	0.12 ± 0.48 E	20.94 ± 0.24 B,C	1.48 ± 1.16 NA	19.46 ± 1.16 A,B,C	0 C
3BS2	57	42	40.32 ± 1.20 A,B,C,D	0.04 ± 0.28 E	20.98 ± 0.14 B	1.64 ± 1.16 NA	19.34 ± 1.71 A,B,C	0 C
3BS4	55	42	39.78 ± 1.53 B,C,D	0.04 ± 0.2 E8	20.98 ± 0.14 B	2.18 ± 1.48 D	18.80 ± 1.50 B,C,D	0 C
3BS1	33	42	40.20 ± 0.97 A,B,C,D	0.04 ± 0.28 E	20.98 ± 0.14 B	1.76 ± 0.96 NA	19.22 ± 0.95 A,B,C	0 C
3BS5	7	42	39.64 ± 1.50 B,C,D	0.08 ± 0.57 E	20.96 ± 0.28 B	2.28 ± 1.34 D	18.68 ± 1.39 B,C,D	0 C
3BL2	22	42	34.00 ± 2.48 I,J,K	2.20 ± 1.91 B	19.90 ± 0.95 F,G,H,I,J,K	5.80 ± 1.84 A,B	14.10 ± 1.92 H,I	0 C
3BL8	28	42	37.54 ± 1.84 E,F,G,H	0.36 ± 0.78 D,E	20.82 ± 0.39 B,C,D,E	4.10 ± 1.69 B,C,D	16.72 ± 1.73 E,F,G	0 C
3BL1	31	42	36.80 ± 2.16 G,H,I,J	1.22 ± 1.11 B,C	20.20 ± 0.83 E,F,G,H,I,J	4.52 ± 1.95 B,C	15.68 ± 2.03 F,G,H	0.46 ± 0.50 A,B
3BL9	38	41	33.10 ± 2.38 J,K	2.18 ± 1.51 A,B	19.38 ± 0.83 K,L	5.70 ± 1.82 A,B	13.68 ± 2.00 H,I	0.02 ± 0.14 C
3BL10	50	42	35.90 ± 2.50 H,I,J	1.04 ± 1.47 C,D	20.44 ± 0.76 B,C,D,E,F,G	5.04 ± 2.02 B	15.40 ± 2.15 F,G,H	0.02 ± 0.14 C
3BL7	63	41	33.46 ± 2.57 J,K	2.12 ± 1.22 A,B	19.44 ± 0.61 K,L	5.42 ± 2.13 A,B	14.02 ± 2.28 G,H,I	0 C

**Table 2:** Results of expression for the genes assigned to chromosome 3B. Number of genes expressed during meiosis assigned to the different deletion bins of chromosome 3B. Estimated size (Mb): estimated size of each deletion bin; Number of genes mapped in bins: Total number of high confidence (HC) genes mapped per bin; Number of genes expressed: Number of genes expressed (FPKM > 5) per bin; Percentage of genes expressed: associated percentage of expressed genes; Number of genes on 3B overexpressed: number of genes for which the 2^^log2^ (Fold Change FPKM) is >2 in both group 3 B–A and B–D comparisons. Genes without homoeologous copies were included.

Arm	Deletion Bin	Estimated Size (Mb)	Number of Genes Mapped in Bins	Number of Genes Expressed	Percentage of Genes Expressed	Number of Genes on 3B Overexpressed
Short	3BS3-0.87-1.00	4.46	93	28	30.11	7
	3BS8-0.78-0.87	31.38	519	100	19.27	23
	3BS7-0.75-0.78	5.93	58	18	31.03	3
	3BS2-0.57-0.75	75.56	644	189	29.35	38
	3BS4-0.55-0.57	20.31	117	47	40.17	8
	3BS1-0.33-0.55	105.52	608	241	39.64	43
	3BS5-0.07-0.33	53.08	149	85	57.05	12
	C-3BS5-0.07	48.04	67	38	56.72	11
Long	C-3BL2-0.22	90.60	416	223	53.61	40
	3BL2-0.22-0.28	28.46	189	67	35.45	19
	3BL8-0.28-0.32	17.85	106	48	45.28	11
	3BL1-0.32-0.38	43.64	260	124	47.69	16
	3BL9-0.38-0.50	44.96	359	164	45.68	25
	3BL10-0.50-0.63	48.48	314	144	45.86	26
	3BL7-0.63-1.00	204.93	2161	583	26.98	115
Total Short Assigned		344.28	2255	746	33.08	145
Total Long Assigned		478.92	3805	1353	35.56	252
Total Assigned		823.20	6060	2099	34.64	397

**Table 3:** Known meiotic genes assigned to chromosome 3B. Meiotic gene detected using model species protein (Arabidopsis) and exonerate alignment (score >500 identity >40% similarity >60%). Gene name determined according to Arabidopsis. Gene ID: ID of 3B-homoeologous copy gene in *Triticum aestivum* annotation. Deletion bin: Deletion bin where the gene is assigned. FPKM Values (average of four meiotic stages and two replicates [55]) are indicated for the three homoeologous copies when available; the most expressed copy is in bold.

Related	Deletion Bin	Gene ID	FPKM3A	FPKM3B	FPKM3D
BRCA2	3BS2-0.57-0.75	TraesCS3B02G115500	**5.26 ± 2.72**	1.75 ± 0.99	0.93 ± 0.51
MSH7	3BS4-0.55-0.57	TraesCS3B02G136600	**4.13 ± 2.54**	3.08 ± 1.76	3.37 ± 1.61
DUO1-3S	3BS1-0.33-0.55	TraesCS3B02G178200	0.45 ± 0.19	0.74 ± 0.39	**7.37 ± 4.01**
CYCA1;2/TAM1	3BS1-0.33-0.55	TraesCS3B02G183400		**24.66 ± 7.16**	
MUS81-3S	3BS5-0.07-0.33	TraesCS3B02G218300	6.29 ± 3.17	6.41 ± 3.93	**21.67 ± 7.85**
RTEL1	C-3BL2-0.22	TraesCS3B02G242700	**5.87 ± 2.53**	2.92 ± 1.55	2.29 ± 1.26
DUO1-3L	C-3BL2-0.22	TraesCS3B02G254800	**4.77 ± 2.45**	4.07 ± 2.52	3.30 ± 1.77
MPK4-1	C-3BL2-0.22	TraesCS3B02G256700	**21.08 ± 7.62**	9.83 ± 3.93	7.61 ± 4.64
MPK4-2	C-3BL2-0.22	TraesCS3B02G260900	4.07 ± 2.39	4.47 ± 2.64	**7.44 ± 3.32**
MPK4-3	C-3BL2-0.22	TraesCS3B02G270200	13.01 ± 7.36	**17.93 ± 8.58**	14.06 ± 7.12
ASK1/ASK2	3BL1-0.32-0.38	TraesCS3B02G308600			
CAP-E1/E2	3BL7-0.63-1.00	TraesCS3B02G423800	11.63 ± 4.45	**18.08 ± 9.11**	11.23 ± 6.71
SYN4	3BL7-0.63-1.00	TraesCS3B02G429700	20.58 ± 9.22	16.23 ± 9.71	**31.22 ± 8.59**
ZIP4	3BL7-0.63-1.00	TraesCS3B02G434600	5.79 ± 2.94	5.91 ± 3.28	**6.07 ± 2.13**
MUS81-3L	3BL7-0.63-1.00	TraesCS3B02G535000	9.96 ± 6.53	19.29 ± 3.30	**36.59 ± 11.18**
MLH1*	3BL7-0.63-1.00	TraesCS3B02G564100	3.90 ± 1.89	**13.29 ± 4.19**	12.06 ± 4.84

* There are two copies of *Mlh1* gene on chromosome 3D, and the expression value is a mean of the two.

## Data Availability

All raw data are provided in Table S1.

## Supplementary Materials

The following supporting information can be downloaded at https://www.mdpi.com/article/10.3390/plants11172281/s1: Figure S1: Scatterplots of the number of chiasmata, univalents, bivalents, rods, rings and multivalents for all the deletion and aneuploid lines; Table S1: Raw data of the number of chiasmata, univalents, bivalents, rods, rings and multivalents for all the deletion and aneuploid lines; Table S2: Normality tests (Shapiro–Wilk) of the number of chiasmata, univalents, bivalents, rods, rings and multivalents for all the deletion and aneuploid lines; Table S3: Kruskal–Wallis tests of the number of chiasmata, univalents, bivalents, rods, rings and multivalents for all the deletion and aneuploid lines; Table S4: Mann–Whitney tests of the number of chiasmata, univalents, bivalents, rods, rings and multivalents for all the deletion and aneuploid lines. Table S5: List of genes present on chromosome 3B; analysis of those that are over-expressed; expression of those known to be involved in recombination; Table S6: Gene onthology terms; Table S7: ID and protein sequences (Arabidopsis) of the genes known as involved in recombination pathway; Table S8: Comparative analyses of the different homoeologous copies (proteins) of the putative candidate genes to assess for their functionality.
