# Supplementary material for: Location and Identification on Chromosome 3B of Bread Wheat of Genes Affecting Chiasma Number
Source: Plants (Basel). 2022 Aug 31;11(17):2281. doi: 10.3390/plants11172281 (PMC9460588; doi:10.3390/plants11172281)
Supplement: Supplementary file 1 [file plants-11-02281-s001.zip › FigS1-Darrier-et-al-2022.pptx]

## Slide 1
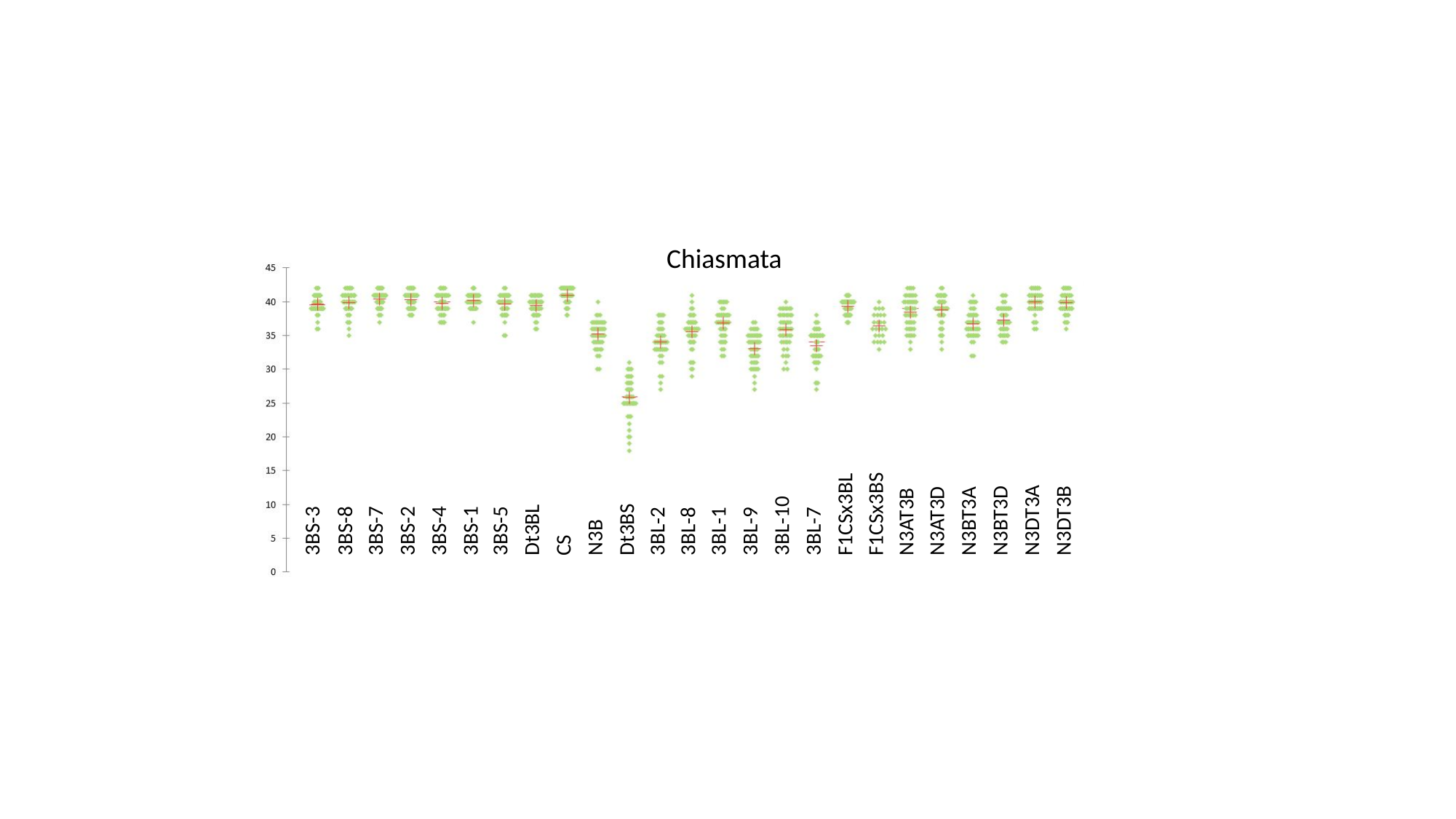

Chiasmata
F1CSx3BS
F1CSx3BL
N3DT3A
N3DT3B
N3BT3D
N3AT3D
N3BT3A
N3AT3B
3BL-10
Dt3BS
Dt3BL
3BS-3
3BS-8
3BS-7
3BS-2
3BS-4
3BS-1
3BS-5
3BL-2
3BL-8
3BL-1
3BL-9
3BL-7
N3B
CS

## Slide 2
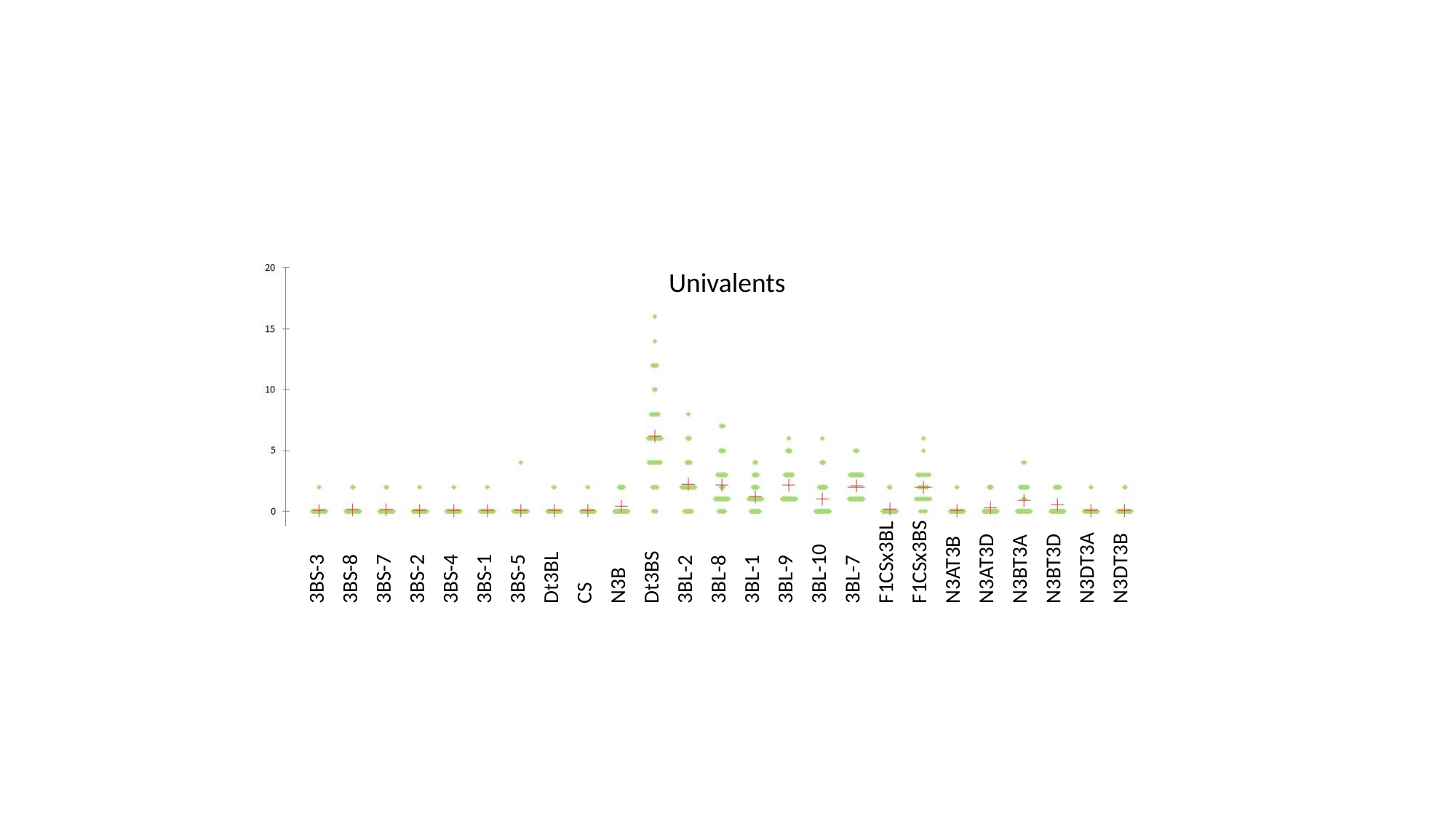

Univalents
F1CSx3BS
F1CSx3BL
N3DT3A
N3DT3B
N3BT3D
N3AT3D
N3BT3A
N3AT3B
3BL-10
Dt3BS
Dt3BL
3BS-3
3BS-8
3BS-7
3BS-2
3BS-4
3BS-1
3BS-5
3BL-2
3BL-8
3BL-1
3BL-9
3BL-7
N3B
CS

## Slide 3
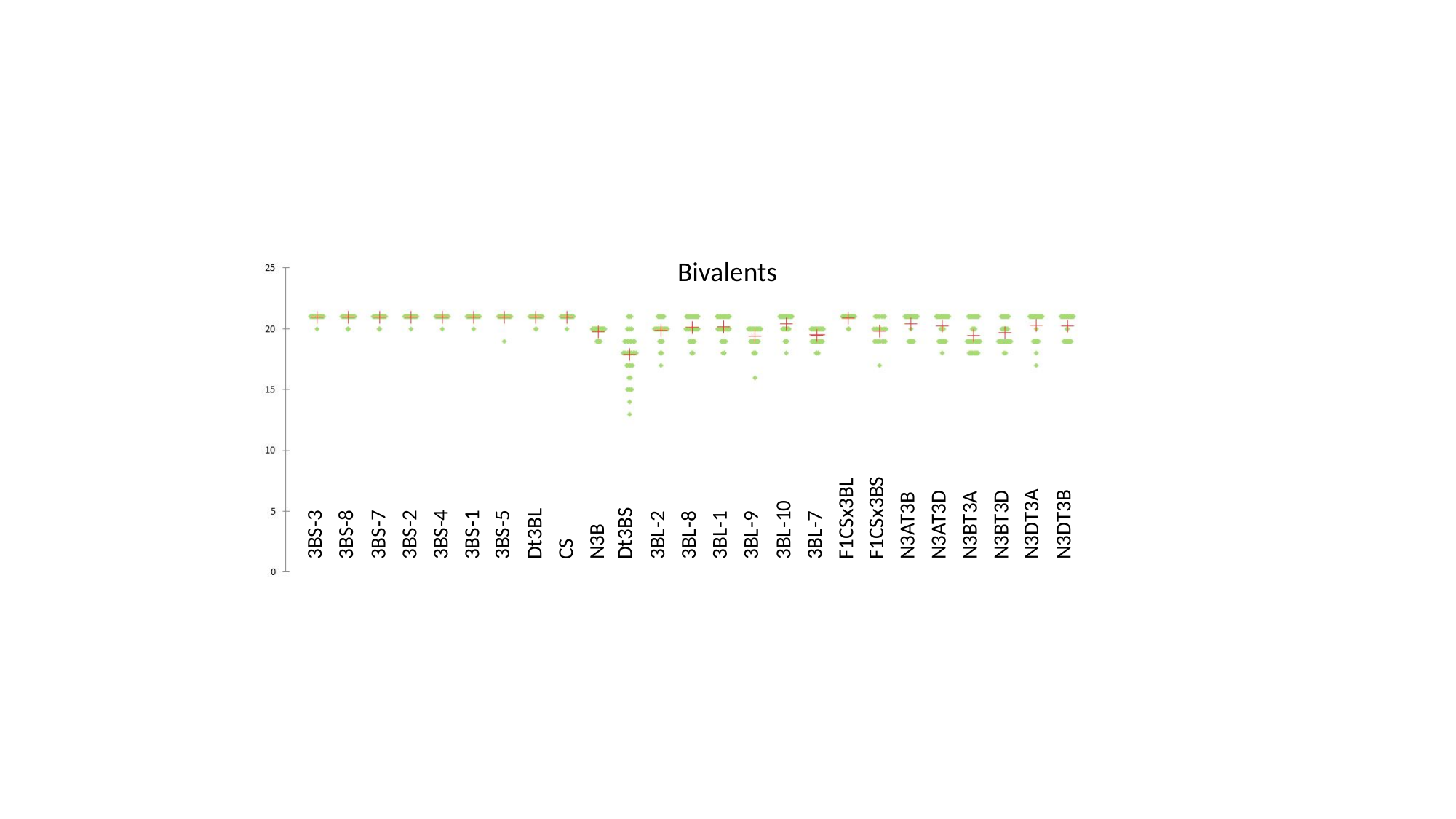

Bivalents
F1CSx3BS
F1CSx3BL
N3DT3A
N3DT3B
N3BT3D
N3AT3D
N3BT3A
N3AT3B
3BL-10
Dt3BS
Dt3BL
3BS-3
3BS-8
3BS-7
3BS-2
3BS-4
3BS-1
3BS-5
3BL-2
3BL-8
3BL-1
3BL-9
3BL-7
N3B
CS

## Slide 4
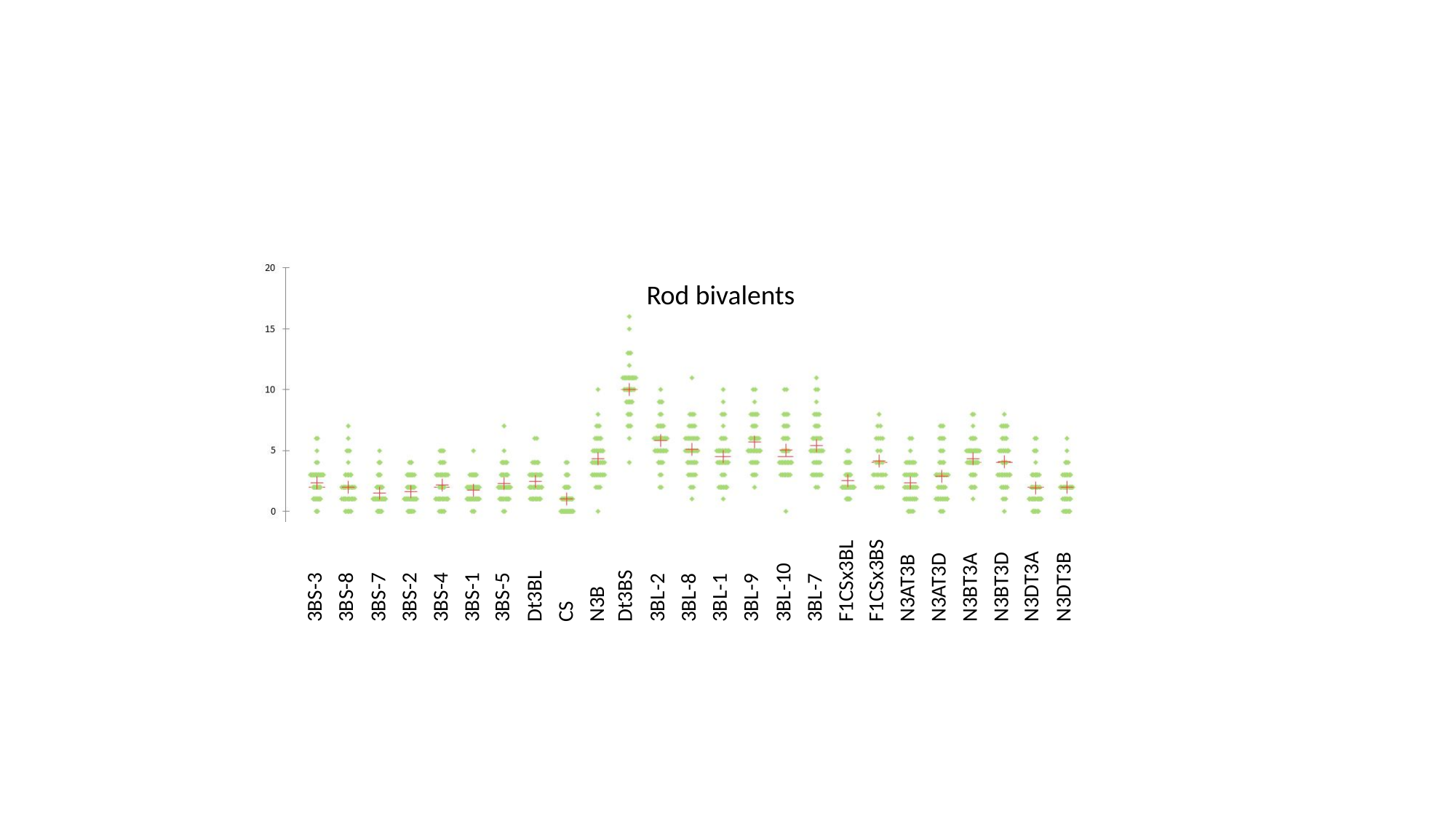

Rod bivalents
F1CSx3BS
F1CSx3BL
N3DT3A
N3DT3B
N3BT3D
N3AT3D
N3BT3A
N3AT3B
3BL-10
Dt3BS
Dt3BL
3BS-3
3BS-8
3BS-7
3BS-2
3BS-4
3BS-1
3BS-5
3BL-2
3BL-8
3BL-1
3BL-9
3BL-7
N3B
CS

## Slide 5
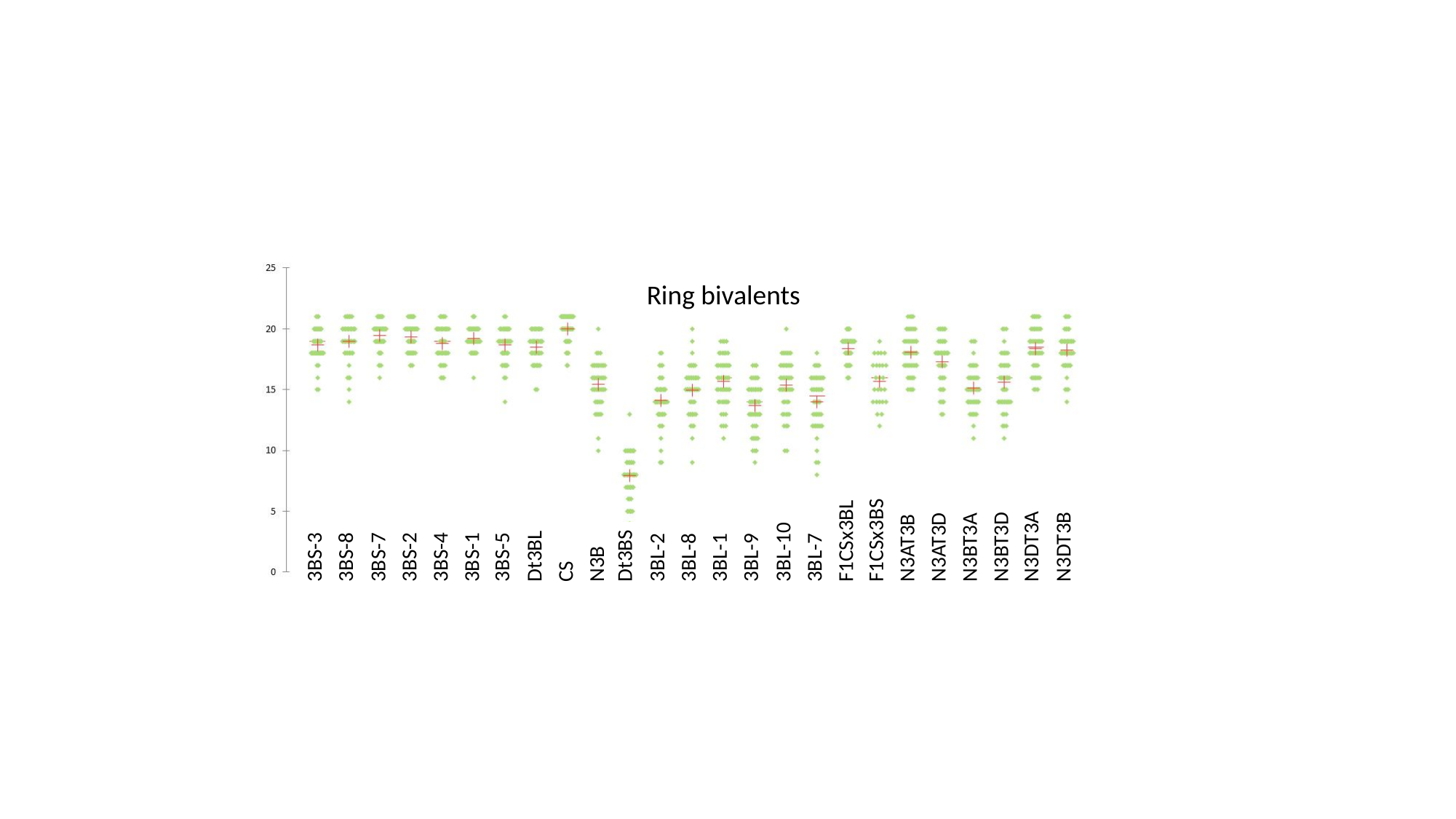

Ring bivalents
F1CSx3BS
F1CSx3BL
N3DT3A
N3DT3B
N3BT3D
N3AT3D
N3BT3A
N3AT3B
3BL-10
Dt3BS
Dt3BL
3BS-3
3BS-8
3BS-7
3BS-2
3BS-4
3BS-1
3BS-5
3BL-2
3BL-8
3BL-1
3BL-9
3BL-7
N3B
CS

## Slide 6
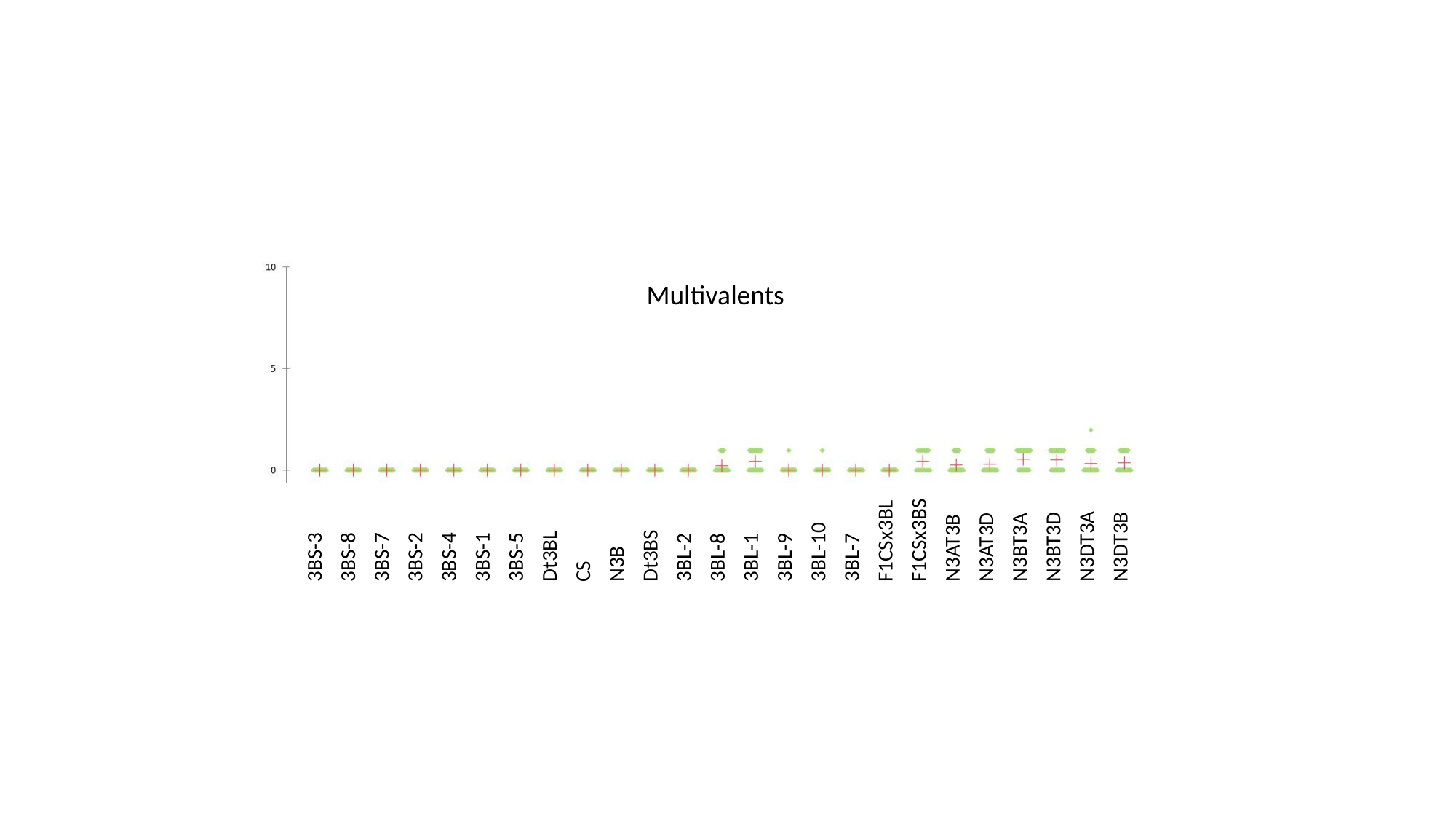

Multivalents
F1CSx3BS
F1CSx3BL
N3DT3A
N3DT3B
N3BT3D
N3AT3D
N3BT3A
N3AT3B
3BL-10
Dt3BS
Dt3BL
3BS-3
3BS-8
3BS-7
3BS-2
3BS-4
3BS-1
3BS-5
3BL-2
3BL-8
3BL-1
3BL-9
3BL-7
N3B
CS
